# Brazilian version of the Rivermead Post-Concussion Symptoms Questionnaire

**DOI:** 10.1590/0004-282X-ANP-2020-0273

**Published:** 2021-05-01

**Authors:** Marcia Mitie Nagumo, Renata Eloah de Lucena Ferretti-Rebustini, Marcos Alencar Abaide Balbinotti, Daniele Vieira da Silva, Cintya Yukie Hayashi, Wellingson Silva Paiva, Manoel Jacobsen Teixeira, Robson Luis Oliveira de Amorim

**Affiliations:** 1 Universidade de São Paulo Faculdade de Medicina Departamento de Neurologia São Paulo SP Brazil Universidade de São Paulo, Faculdade de Medicina, Departamento de Neurologia, São Paulo SP, Brazil.; 2 Universidade de São Paulo Escola de Enfermagem Programa de Pós-Graduação em Enfermagem na Saúde do Adulto São Paulo SP Brazil Universidade de São Paulo, Escola de Enfermagem, Programa de Pós-Graduação em Enfermagem na Saúde do Adulto, São Paulo SP, Brazil.; 3 Université du Québec à Trois-Rivières Department of Psychology Québec QC Canada Université du Québec à Trois-Rivières, Department of Psychology, Québec QC, Canada.; 4 Universidade Federal do Amazonas Manaus AM Brazil Universidade Federal do Amazonas, Manaus AM, Brazil.

**Keywords:** Brain Injuries, Traumatic, Post-Concussion Syndrome, Translating, Psychometrics, Lesões Encefálicas Traumáticas, Síndrome Pós-Concussão, Tradução, Psicometria

## Abstract

**Background:**

After a traumatic brain injury, post-concussion symptoms are commonly reported by patients. Although common, these symptoms are difficult to diagnose and recognize. To date, no instruments evaluating post-concussion symptoms have been culturally translated or adapted to the Brazilian context.

**Objective:**

To culturally adapt the Rivermead Post-Concussion Symptoms Questionnaire for use in Brazilian Portuguese.

**Methods:**

Cross-cultural adaptation was done in five steps: translation, synthesis of translations, back-translation, evaluation by two expert committees and two pretests among adults in a target population.

**Results:**

The semantic, idiomatic, cultural and experimental aspects of the adaptation were considered adequate. The content validity coefficient of the items regarding language clarity, pratical pertinence, relevance and dimensionality were considered adequate for evaluating the desired latent variable. Both pretests demonstrated that the instrument had satisfactory acceptability.

**Conclusion:**

The Brazilian version, named Questionário Rivermead de Sintomas pós Concussionais (RPQ-Br), has been adapted, and is ready for use in the Brazilian context.

## RESUMO

**Introdução:**

Após um traumatismo cranioencefálico, os sintomas pós-concussionais são comumente relatados pelos pacientes e, embora comuns, são difíceis de serem reconhecidos. Até o momento, não existem instrumentos que tenham sido traduzidos ou adaptados culturalmente para o contexto brasileiro.

**Objetivo:**

Adaptação transcultural do *Rivermead Post Concussion Symptoms Questionnaire* para o português do Brasil.

**Métodos:**

O processo de adaptação transcultural foi realizado em cinco etapas: tradução, síntese das traduções, retrotradução, avaliação por dois comitês de especialistas e dois pré-testes com adultos da população-alvo.

**Resultados:**

Os aspectos semânticos, idiomáticos, culturais e experimentais foram considerados adequados. Os coeficientes de validade de conteúdo dos itens quanto à clareza da linguagem, pertinência teórica, relevância prática e dimensionalidade foram considerados adequados para avaliar a variável latente desejada. Ambos os pré-testes demonstraram aceitabilidade satisfatória do instrumento.

**Conclusão:**

A versão brasileira do Questionário Rivermead de Sintomas pós Concussionais (RPQ-Br) foi traduzida e adaptada com sucesso e está pronta para ser utilizada no contexto brasileiro.

## INTRODUCTION

Traumatic brain injury (TBI) is characterized by any injury from an external trauma resulting in anatomical changes to the skull, such as fracture or laceration of the scalp, or functional impairment of the meninges, the brain or its vessels, with consequent temporary or permanent cerebral changes of cognitive or functional nature^1^.

According to the Centers for Disease Control and Prevention approximately 1.7 million people in the United States suffer TBI annually^2^. Although epidemiological studies are scarce in Latin American countries, it has been estimated that in Brazil there are around 125,000 hospital admissions due to TBI per year, with an incidence of 65.7 admissions per 100,000 inhabitants and a hospital mortality rate of 5.1/100,000/year^3^. In European studies, an annual incidence of 250/500-100,000 TBI cases has been reported, and the majority (90%) were considered to be mild TBI^4^.

Classification of TBI severity can be done based on the Glasgow Coma Scale^5^ (GCS). The GCS is a point scale that is used to assess a patient's level of consciousness and neurological functioning after a brain injury. The score is based on the best eye-opening response (1‒4 points), best motor response (1‒6 points) and best verbal response (1‒5 points). Patients with mild TBI will have a score between 13‒15 points, those with moderate TBI will have 9‒12 points and those with severe TBI will have <9 points. While some patients do not present any symptoms after mild or moderate TBI, approximately 50% will experience a variety of post-concussion symptoms^6^.

Post-concussion syndrome (PCS) is the term used to describe a set of often disabling symptoms that occur after TBI, even when there is no detectable intracranial lesion on imaging tests^7^. Post-concussion symptoms can be physical, cognitive, emotional or behavioral, such as headache, dizziness, nausea, changes in coordination and balance, changes in appetite, sleep, vision and hearing, fatigue, anxiety, depression, irritability or problems with memory, concentration and decision-making^8^.

To date, no instrument evaluating PCS has been created, adapted or validated for the Brazilian context. Thus, the purpose of this study was to perform cross-cultural adaptation of the Rivermead Post-Concussion Symptoms Questionnaire for use in Brazilian Portuguese, to assess patients with mild or moderate TBI.

## METHODS

This was a cross-cultural adaptation process (CCA) conducted on a health measurement instrument that followed the procedures proposed by Beaton et al.^9^. Firstly, the author of the original scale was approached and granted permission for the transcultural adaptation and scale validation, for use in Brazil, to be performed. Before the study was started, it was approved by the Research Ethics Committee at the Hospital das Clínicas of the University of São Paulo School of Medicine, in São Paulo, Brazil.

All participants, in all steps, received detailed verbal and written instructions about the study and agreed to participate by signing a commitment statement (translators and specialists) or an informed consent statement (patients).

### Instrument

The Rivermead Post-Concussion Symptoms Questionnaire (RPQ)^10^ was created in England and contains a list of 16 symptoms that frequently occur in individuals post-TBI. It evaluates the severity of the symptoms over the last 24 hours prior to applying the instrument. The symptoms evaluated are the following: headache, dizziness, nausea or vomiting, sensitivity to noise and light, sleep disturbances, fatigue, irritability, changes to memory, double vision, blurred vision, restlessness, difficulty concentrating, feeling frustrated and/or impatient and/or depressed and/or tearful. To assess the severity of each symptom, the instrument has a Likert-type^11^ scale, on which a rating of zero represents “not experienced at all” and a rating of four represents “a severe problem”. The final score consists of the sum of the patient's ratings and can range from zero to 64 points. The instrument can be applied in interview form or can be self-applied.

The RPQ is a widely used tool for assessing the presence and severity of various post-concussion symptoms. Assessment of these symptoms at an early post-injury or follow-up stage may allow prediction of clinical trajectories and help to guide treatments.

### Cross-cultural adaptation process of the instrument

**Figure 1:**
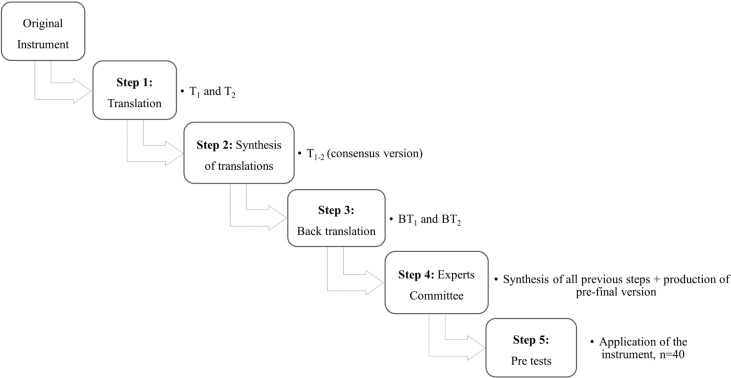
Flowchart of the cross-cultural process of the Rivermead Post-Concussion Symptoms Questionnaire. São Paulo, 2018.

The CCA process consisted of the following steps^9^: 1) translation; 2) synthesis of translations; 3) back-translation; 4) evaluation by expert committees; and 5) pretests. Figure 1 illustrates the flowchart of the CCA process.

The translation of the original instrument from English to Brazilian Portuguese was performed by two independent bilingual translators, both native to Brazil. The first translator (T1) had medical training and knowledge of the study objectives and the original instrument. The second translator (T2) had no previous medical background and had no knowledge of the study objectives or connection with the study field.

The two translations (T1 and T2) were combined into a single version (T1-2) that was created by a third independent translator (T3), a bilingual Brazilian native with previous medical training. The objective of this step was to produce a consensus version that reflected the agreement of the two previous translations and standardized the use of divergent expressions.

Subsequently, two back translations (BT1 and BT2) were performed. These were carried out by two new independent translators (T4 and T5), who were native English speakers, fluent in the Brazilian Portuguese language, without any previous knowledge of the original instrument. The objective of this step was to ascertain the consistency of the translations performed in the first step of the CCA process.

All the material from the previous steps (T1, T2, T1-2, BT1, BT2 and the original instrument) was then handed over to a multidisciplinary expert committee (EC) composed of a psychometrist, a neurologist, a neurosurgeon, a nurse, a neuropsychologist and a Portuguese language teacher. All members of this committee were bilingual Brazilian natives, with extensive knowledge or experience in the area of psychometry and/or cognition and/or neurology and/or TBI.

The purpose of this EC was to analyze the adequacy of the translation and back translation, in order to determine whether the semantic, idiomatic, cultural and conceptual equivalences had been maintained. The EC was asked to review the translation of the instrument into Portuguese and demonstrate whether they agreed with the translation, by using an equivalence scale proposed by Waltz, Strickland and Lenz^12^: −1 (not equivalent), zero (undecided) and +1 (equivalent). Additionally, they were permitted to give suggestions to improve the translation, if necessary. The EC could modify and eliminate items that they considered irrelevant, inadequate or ambiguous, and could also propose substitutes that applied to situations in local (Brazilian) culture, while always maintaining the same general concept as in the item that was replaced.

Once the EC had performed their analysis, the research team responsible for conducting the study met to review all the procedures performed, protect the adequacy of the CCA process, develop a pre-final version, analyze any discrepancies and reach a consensus for decision-making. This team was composed of a nurse with experience in neurotrauma; a PhD professor in nursing and with experience in neuroscience and psychometry; and a neurosurgeon who was a professor of neurology and neurotrauma.

Upon completion of the CCA process, content validation of the instrument was performed. To evaluate the content, a second EC was formed, which consisted of two neurosurgeons, a neurologist and a neuropsychologist. None of these members had participated in the first EC, or in any previous steps of the CCA process. The objective of this EC was to evaluate the following areas: language clarity, practical relevance, theoretical relevance and dimensionality of each of the items in the instrument. This evaluation was done using a Likert-type scale from 1 to 5, where 1 represented “very little clarity/of very little pertinence/of very little relevance/not very representative of the phenomena” and 5 represented “very clear/very pertinent/very relevant/very representative of the phenomena”, following the previously established theoretical principles^11^.

### Procedures with patients

The understandability and acceptability of the instrument, as well as the clarity of the instructions and the items listed in the questionnaire, were verified in the first pretest. Ten participants from the target population were questioned about the comprehension difficulties and importance of each item in relation to their own situation. Several final adjustments were necessary due to difficulties reported by these patients.

The pre-final version was applied to a convenience sample of 30 patients^9^. Individuals from the target population were recruited at the Central Institute's emergency room or the Neurotrauma Outpatient Clinic of the University of São Paulo's healthcare system. The patient selection met the following criteria:

- Inclusion criteria: 18 years or older, Brazilian, of any gender, race and ethnicity; history of mild or moderate TBI according to the Glasgow Coma Scale^5^ upon hospital admission; post-TBI time of between three and 24 months at the time of evaluation; and Brazilian Portuguese as first language.
- Exclusion criteria: Inability to communicate with researchers, thus making it difficult to apply the instrument; and presence of a pre-morbid psychiatric diagnosis (schizophrenia, dementia or bipolar disorder), either documented in the patient's medical record or reported by the patient/relative/legal guardian.

### Statistical analyses

All questionnaires were administered on printed paper and later tabulated in an Excel spreadsheet (Microsoft Excel for Mac 2011, version 14.7.2).

For the descriptive analysis, the continuous/discrete quantitative variables were presented as the mean and standard deviation or the median and interquartile range, according to the distribution. The ordinal and categorical variables were presented as the absolute and relative frequency.

In order to evaluate the content during the first EC, the content validity coefficient (CVC)^13^ was applied, using the PABAK (prevalence-adjusted, bias-adjusted kappa) test to rule out the possibility of response prevalence bias between the evaluators. In the second EC, the principles proposed by Hernández-Nieto^14^ were used to calculate the CVC. Different calculations were used because the two ECs conducted during the study had different goals. The main goal of the first EC was to evaluate translation quality and Portuguese-language adequacy, while the second EC evaluated the instrument adequacy in the TBI domain.

All tests were two-tailed and p<0.05 were considered statistically significant. The analyses were performed using the Statistical Package for the Social Sciences (SPSS) software, version 21.0 (SPSS, Inc., Chicago, Illinois, USA) for Mac.

## RESULTS

The CCA process resulted in a synthesis of translations, two back translations and one pre-final version (Table 1). In the back translation step, the two versions presented (BT1 and BT2) were very similar to the original instrument. The concordance of the six evaluators in the first EC was analyzed with regard to each of the four types of equivalence. Table 2 shows that the CVI values were higher than 85% and that all types of equivalence reached statistically significant values (p<0.001), with moderate to almost perfect concordance strength^15^.

**Table 1:** Original instrument, synthesis of translations, back-translations and pre-final version of the Brazilian Version of the Rivermead Post-Concussion Symptoms Questionnaire. São Paulo, 2018.

Original Instrument	Synthesis of translations	Back-translation 1	Back-translation 2	Pre-final version
Rivermead Post-Concussion Symptoms Questionnaire	Questionário Rivermead de Sintomas Pós-Concussionais	Rivermead Questionnaire of Post-Concussion Symptoms	Rivermead questionnaire of post-concussion symptoms	Questionário Rivermead de Sintomas Pós-Concussionais – RPQ-Br
After a head injury or accident some people experience symptoms which can cause worry or nuisance.	Após um ferimento na cabeça ou um acidente, algumas pessoas apresentam sintomas que podem causar preocupação ou incômodo	After a head injury or an accident, some people present symptoms that may cause some concern or discomfort.	After a head injury or accident, some people show symptoms that could cause concern or discomfort	Após um traumatismo na cabeça ou um acidente, algumas pessoas apresentam sintomas que podem causar preocupação ou incômodo.
We would like to know if you now suffer from any of the symptoms given below.	Gostaríamos de saber se você sofre no momento de qualquer um dos sintomas citados abaixo.	We would like to know if you have been experiencing some of the symptoms listed below.	We would like to know if you, at the moment, suffer of any of the symptoms mentioned below	Gostaríamos de saber se, no momento, você apresenta algum dos sintomas citados abaixo.
As many of these symptoms occur normally, we would like you to compare yourself now with before the accident.	Como muitos desses sintomas normalmente ocorrem, gostaríamos que você comparasse sua situação atual com a anterior ao acidente.	Since many of these symptoms usually occur under other circumstances, we would like to compare your present situation to the one before the accident or head injury.	Since many of these symptoms normally happen, we would like you to compare your current situation with that one before the accident.	Como muitos desses sintomas normalmente ocorrem, gostaríamos que você comparasse sua situação atual com a anterior ao acidente.
For each one, please circle the number closest to your answer.	Por favor, para cada pergunta circule o número que mais se aproxima da sua resposta.	Please, for each question, circle the number that most closely matches your answer.	Please, for each question circle the number which gets closer to your answer.	Por favor, para cada pergunta circule o número que mais se aproxima da sua resposta.
0=Not experienced at all	0=Nunca experimentado	0=Never experimented	0=Never experimented	0=Nunca senti
1=No more of a problem	1=Não é um problema	1=This is not a problem	1=It is not a problem	1=Não é mais um problema
2=A mild problem	2=Um problema leve	2=A mild problem	2=A mild problem	2=É um problema leve
3=A moderate problem	3=Um problema moderado	3=A moderate problem	3=A moderate problem	3=É um problema moderado
4=A severe problem	4=Um problema grave	4=A severe problem	4=A severe problem	4=É um problema grave
Compared with before the accident, do you now (i.e., over the last 24 hours) suffer from:	Comparado a antes do acidente, você agora (ao longo das últimas 24 horas) sofre de:	Compared to before the accident, have you experienced in the last 24 hours:	Compared to before the accident, you now (for the last 24 hours) are suffering of:	Comparando a antes do acidente, você (nas últimas 24 horas) apresenta:
Headaches	Dores de cabeça	Headaches	Headaches	Dores de cabeça
Feelings of Dizziness	Sensações de tontura	Dizziness	Dizziness	Sensações de tontura
Nausea and/or Vomiting	Náusea e/ou vômito	Nausea and/or vomiting	Nausea and/or vomiting	Nausea e/ou vômito
Noise Sensitivity, easily upset by loud noise	Sensibilidade ao barulho, incomoda-se facilmente com barulho alto	Sensitivity to noise: you feel easily bothered with loud noises	Susceptibility to noise, get bothered easily by high pitch noise	Sensibilidade ao barulho, incomoda-se facilmente com barulho
Sleep Disturbance	Distúrbios no sono	Sleep disorders	Sleeping disorders	Alteração do sono
Fatigue, tiring more easily	Fadiga, se sente cansado com maior facilidade.	Fatigue, feels tired more easily	Fatigue, gets tired faster	Fadiga, sente-se cansado com maior facilidade
Being Irritable, easily angered	Irritável, facilmente nervoso	Irritability, easily nervous	Irritable, gets nervous easily	Irritação, facilmente irritado
Feeling Depressed or Tearful	Sensação de depressão ou choroso	Feeling of depression or tearfulness	Sensation of depression or tearful	Depressão ou choro fácil
Feeling Frustated or Impatient	Sensação de frustração ou impaciência	Feeling of frustration or impatience	Sensation of frustration or lack of patience	Sensação de frustação ou impaciência
Forgetfulness, poor memory	Esquecimento, memória fraca	Forgetfulness, poor memory	Forgetful, weak memory	Esquecimento, memória ruim
Poor Concentration	Falta de concentração	Lack of concentration	Lack of concentration	Dificuldade de concentração
Taking Longer to Think	Levando mais tempo para pensar	Taking longer to think	Taking more time to think	Lentidão de pensamento
Blurred Vision	Visão turva	Blurred Vision	Blurred vision	Visão embaçada
Light Sensitivity, Easily upset by bright light	Sensibilidade à luz, incomoda-se facilmente com a claridade	Sensitivity to light: you feel easily bothered with brightness	Sensibility to light, gets uncomfortable with brightness	Sensibilidade à luz, incomoda-se facilmente com a claridade
Double Vision	Visão dupla	Double vision	Double vision	Visão dupla
Restlessness	Agitação	Anxiety	Agitation	Inquietação, dificuldade de ficar parado
Are you experiencing any other difficulties?	Você tem vivenciado qualquer outra dificuldade?	Have you experienced any other difficulty?	Have you experienced any other difficulty?	Você apresenta quaisquer outras dificuldades?

**Table 2:** Content validity coefficient and prevalence-adjusted, bias-adjusted Kappa, expert committee, São Paulo, 2018.

	CVC	PABAK	p-value
Semantic equivalence	0.97	0.90	<0.001
Idiomatic equivalence	0.90	0.65	<0.001
Experimental equivalence	0.87	0.56	<0.001
Conceptual equivalence	0.96	0.85	<0.001

CVC: content validity coefficient; PABAK: prevalence-adjusted, bias-adjusted Kappa.

In the second EC, the concordance of the four evaluators was analyzed in relation to each of the domains: clarity, relevance, practical pertinence and dimensionality. Table 3 shows the CVC values of the items.

**Table 3:** Content validity coefficient, expert committee, São Paulo, 2018.

Question	CL	CLPe	RL	RLPe	PP	PPPe	DMS	DMSPe
Q1	1.00	0.003	1.00	0.003	0.95	0.003	0.95	0.003
Q2	1.00	0.003	0.95	0.003	0.95	0.003	0.90	0.003
Q3	0.85	0.003	0.80	0.003	0.80	0.003	0.80	0.003
Q4	0.90	0.003	0.90	0.003	0.90	0.003	0.90	0.003
Q5	0.75	0.003	0.85	0.003	0.90	0.003	0.85	0.003
Q6	0.95	0.003	0.80	0.003	0.80	0.003	0.80	0.003
Q7	0.75	0.003	0.90	0.003	0.95	0.003	0.90	0.003
Q8	0.85	0.003	1.00	0.003	1.00	0.003	1.00	0.003
Q9	0.90	0.003	0.90	0.003	0.90	0.003	0.85	0.003
Q10	0.80	0.003	0.90	0.003	0.85	0.003	0.85	0.003
Q11	0.95	0.003	1.00	0.003	1.00	0.003	0.95	0.003
Q12	0.85	0.003	0.95	0.003	0.95	0.003	1.00	0.003
Q13	0.90	0.003	0.75	0.003	0.70	0.003	0.70	0.003
Q14	1.00	0.003	0.80	0.003	0.80	0.003	0.80	0.003
Q15	0.80	0.003	0.70	0.003	0.70	0.003	0.75	0.003
Q16	0.75	0.003	0.90	0.003	0.90	0.003	0.90	0.003
CVCt	0.872		0.878		0.875		0.865	

CL: clarity; CLPe: clarity error; RL: relevance; RLPe: relevance error; PP: practical pertinence; PPPe: practical pertinence error; DMS: dimensionality; DMSPe: dimensionality error; CVCt: content validity coefficient total.

The four domains showed predominance of good to excellent CVC (CVC>0.70). In the areas of clarity, relevance, practical pertinence and dimensionality (87, 88, 87 and 87%, respectively), the CVC values were all above 0.80. The CVC of the total instrument was 0.87, which confirmed that the instrument has satisfactory content validity for application to the study population.

Regarding the instructions and the list of symptoms of the instrument, the researchers responsible for the study, in conjunction with the EC, chose to change the following words or expressions: from “head injury” (ferimento na cabeça) to “head trauma” (traumatismo na cabeça); the verb “suffer” (sofre) was changed to “presents with” (apresenta); and “never tried” (nunca experimentado) was changed to “never felt” (nunca senti) because we believe that this is a questionnaire that evaluates symptoms and that “never felt” is better understood by the population. “Sleep disturbances” (disturbios do sono) was also modified to “changes in sleep” (alterações no sono) because it is a more adequate term; “feeling of depression” (sensação de depressão) was changed to “depression” (depressão) to make it easier for the patient to understand; “weak memory” (memória fraca) was changed to “poor memory” (memória ruim); “lack of concentration” (falta de concentração) was changed to “difficulty in concentrating” (dificuldade de concentração); “taking more time to think” (levando mais tempo para pensar) was changed to “slowed thinking” (lentidão de pensamento) because it seemed a more adequate term; and, lastly, the verb “you have experienced” (você tem vivenciado) was replaced with “you present with” (você apresenta) for better understanding.

A convenience sample of 10 patients from the target population was selected to participate in the first pretest of the instrument. This sample consisted of 9 men and 1 woman, with a mean age of 47.9±14.87, average schooling (in years) of 9.60±3.68 and with a mean post-trauma time of 9 months. Table 4 shows that there was strong agreement between the individuals with regard to the three questions asked.

**Table 4:** Content validity coefficient and prevalence-adjusted, bias-adjusted Kappa, first pretest, São Paulo, 2018.

	CVC	PABAK	p-value
Does this item apply to you?	1.00	1.00	<0.001
Did you have difficulty understanding this question?	0.95	0.84	<0.001
Did you find the answer options easy to understand?	1.00	1.00	<0.001

CVC: content validity coefficient; PABAK: prevalence-adjusted, bias-adjusted Kappa.

**Figure 2:**
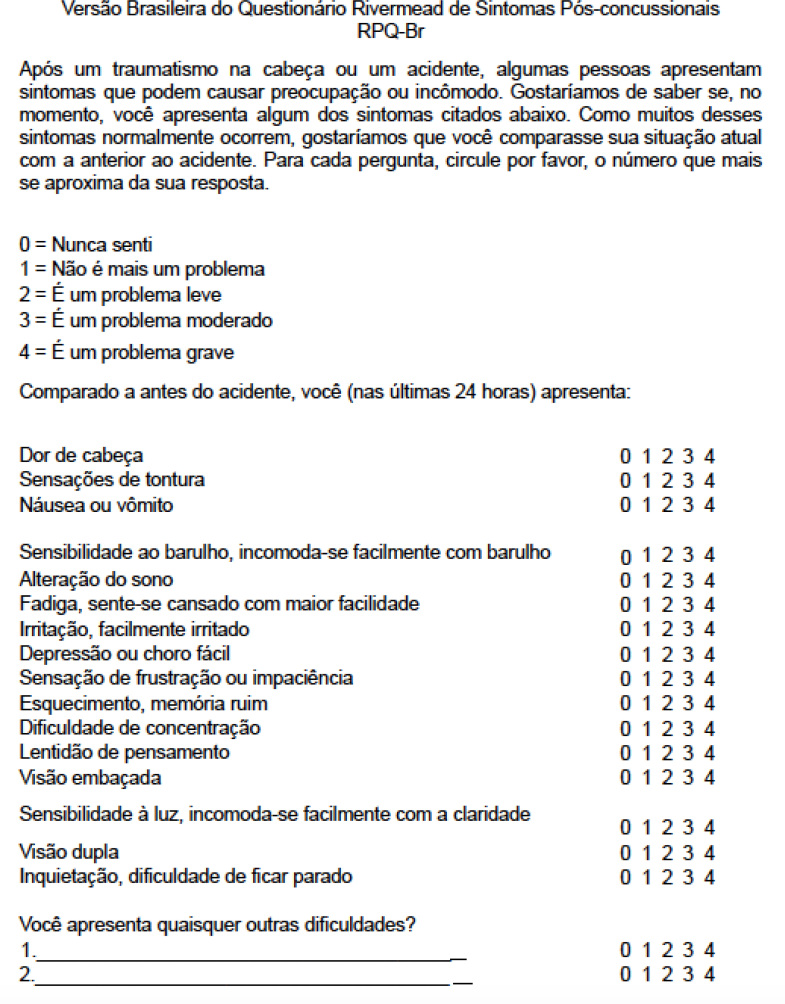
Brazilian Version of the Rivermead Post-Concussion Symptoms Questionnaire: revised pre-final version. São Paulo, 2018.

Despite the strong agreement between the participants, the first pretest resulted in the need to change the following items: from “blurred vision” (visão turva) to “blurry vision” (visão embaçada), from “agitation” (agitação) to “restlessness, difficulty staying still” (inquietação, dificuldade em ficar parado) and from “Compared to before the accident, you now (over the last 24 hours) present with” (Comparado a antes do acidente, você agora (ao longo das últimas 24 horas) apresenta) to “Compared to before the accident, you (in the last 24 hours) present with” (Comparado a antes do acidente, você (nas últimas 24 horas) apresenta), as suggested by the participants. A consensus was reached among the study researchers about the suggestions and notes from the first pretest, and the changes were implemented. The revised pre-final version of the instrument, referred to as the Brazilian version of the Rivermead Post-Concussion Symptoms Questionnaire (RPQ-Br) is presented in Figure 2.

The pretest was given to 30 individuals of the target population. The participants had a mean age of 53±17.5 years, were predominantly male (56%), white (77%), married (53%) and with elementary education (40%). There was a high frequency of mild TBI (83%), and falls were the main mechanism of trauma (63%).

The most prevalent symptoms in the pretest were “forgetfulness, poor memory” (76.7%), “sleep disturbance” (56.7%), “difficulty concentrating” (56.7%) and “slowness of thought” (56.7%).

## DISCUSSION

Adaptation of an instrument is much more complex than simply translating the items of a certain scale. The terms used are not always common to all languages, and cultural variations may change the meaning of what is being measured. Thus, for any instrument to be used in a language other than its original language, it needs to be translated, culturally adapted and, finally, validated^16^. In our study, the cross-cultural adaptation process followed consolidated references and resulted in an adapted version in Brazilian Portuguese with good evidence of content validity.

The ECs were responsible for overseeing all steps of the process to achieve a pre-final instrument that would meet the different types of equivalences. The multidisciplinary ECs played a key role in the CCA process and greatly contributed to the integration of theory and practice. This resulted in an instrument version that was better able to reflect correct cultural adaptation of the original RPQ.

Both pretests were administered to the target population to confirm the clarity and comprehensibility of the instrument. Difficulties and doubts that arose during these steps led to important suggestions regarding how to make the instrument more relatable to the target population. We acknowledge that the first pretest performed in this study was not all-encompassing. The esthetic aspect of the instrument related to quality, and sample size was not explored. Additionally, patients could have been given more specific questions, in order to obtain greater variability of the sample, such as quantifying the importance of each item based on the patients’ conditions, by using a scale from 0 to 100. This may be considered a limitation of the study.

Another point worth noting is that we chose to maintain the format of items that simultaneously evaluated more than one concept. For example, one evaluator noted that each of the items: “nausea and/or vomiting”, “depression or crying easily” and “feelings of frustration or impatience” evaluate two different symptoms in the same answer choice. While this is extremely relevant and pertinent, and the study researchers agreed that these symptoms are different, they decided to maintain the original structure of the instrument so as to not compromise the comparisons of the psychometric analysis (construct validity).

To compare the CCA process of this instrument in relation to other languages and cultures, we performed an extensive search of the literature. However we were unable to encounter anything relevant, which makes it difficult to compare the CCA processes with regard to knowledge of difficulties found in populations different from ours.

Despite the aforementioned limitations, the RPQ-Br is a useful and easy-to-apply tool for multidisciplinary teams who are seeking a more overall post-trauma follow-up and a clearer way of recognizing symptoms when caring for post-TBI patients. The RPQ-Br can be used to follow up the improvement/worsening of and severity of symptoms, to provide a better indication for diagnosing PCS.

Based on our findings, we believe that the steps of the CCA process for the RPQ for Brazilian Portuguese were satisfactorily fulfilled. At the end of this process, we completed an important step so that the translated instrument could be made available for use among post-TBI Brazilian patients. We understand the importance of performing advanced psychometric analyses to ensure the reliability and validity of the final translated instrument. The study investigators already have these validity and reliability analyses, and they will be published in a second paper, for better exploration of this important psychometric assessment.

In conclusion, the Brazilian version of the RPQ (RPQ-Br) has been translated into the Brazilian Portuguese language/culture and provides satisfactory evidence of content validity for evaluation of post-concussion symptoms in patients with mild to moderate TBI.
